# When identity is not reached: two cases from Brasilia

**DOI:** 10.1080/20961790.2022.2076797

**Published:** 2023-02-12

**Authors:** Aluisio Trindade Filho

**Affiliations:** Medicolegal Institute, Civil Police of Federal District, Brasilia, Brazil

**Keywords:** Forensic sciences, forensic anthropology, human identification failure, missing person database, primary methods of identification

## Abstract

When human remains are examined, three questions always need to be answered: who is the deceased, what was the cause of death, and when did the death occur, the former question being the most relevant. The identification of half or fully skeletonized human remains is a complex process and always requires the use of methods that allow individualization beyond any reasonable doubt. However, no matter how vigorous the search for identification, this is not always achieved. Here, the author presents two cases in which identification was exhaustively attempted but not achieved despite the existence of an osteo implanted device in one case and the presence of documents in the other. In one case, we could not find a potential identity for the deceased, while in the other we found a possible identity but not a family member to provide antemortem data to confirm it. Although the scientific literature tends to favour the publication of cases with favourable outcomes, one should also learn from failures, which is the reason why the author decided to publish his unsuccessful experiences. The reasons for the failures are discussed, as well as methodological improvements for future cases.

## Key points

Two cases analysed are both from Brasilia, Brazil.

High initial expectation of achieving identification due to implanted osteo device in one case and presence of documents in the other.

Non-scientific strategies to increase the possibility of identification, such as publication in local media of postmortem data relevant to identification and the active pursuit of antemortem information.

Unsuccessful attempts to contact family members of the deceased.

Suggestions for how to increase the chances of identification in similar future cases.

## Introduction

The central goal of Forensic Anthropology (FA) is to identify the deceased. To achieve this, there are a series of methods and techniques for improving the likelihood of a successful identification. However, even with a complete skeleton and set of characteristics with a high degree of discrimination (e.g. osteo implanted devices, congenital defects, tattoos, and documents, although the latter may only to indicate a way forward), the deceased is not always identified.

The scientific literature tends to favour the publication of successful cases over works that present unsuccessful ones. This type of bias is called publication bias and results from the tendency of researchers and journal editors to accept for publication manuscripts “based on the direction or strength of the study findings” [1], “i.e. the outcomes” [2]. FA literature is no different in this regard. As Francis Bacon said in 1621, “the human intellect … is moved more by affirmations than by negations”.

In this article, the author, a forensic pathologist and forensic anthropologist, presents two of his cases from the Forensic Anthropology Laboratory (LAF), Medicolegal Institute, Civil Police of the Federal District (PCDF), Brazil, which, despite seeming straightforward in the beginning, resulted in failed identifications. The motivation behind the publication of these cases was that similar situations likely occur with all professionals who perform FA identifications, making it beneficial for those in the field to learn from failed cases. This work therefore presents two cases ending in unsuccessful identification, as well as proposals for improving the infrastructure that supports the identification process.

## Cases and methods

The first case involved an incomplete human skeleton found with several bones partially destroyed by the action of taphonomic processes, while the second involved human remains found in an advanced state of decomposition. After receiving the materials and following the internal protocol of the LAF, anthropologists and other staff travelled to the respective sites where the materials of both cases were found, where they recovered more bones and teeth.

For both cases, the bioanthropological profile was obtained using the various methods listed in the most current version of the LAF internal protocol at the time. Sex was established by (1) a metric evaluation of the pelvis according to Murail et al. [3] and (2) a non-metric evaluation of parameters of the skull. The age range was estimated by (1) the evolutionary phase of the medial epiphysis of the clavicle, (2) the annular epiphyses of the vertebral bodies, (3) the articular surface and costal tubercle of the first rib according to DiGangi et al. [4], (4) the articular surface of the fourth rib according to Hartnett [5], and (5) pubic symphysis according to the protocol of Suchey and Brooks modified by Hartnett [6]. Height was evaluated by applying the Trotter and Gleser equations to the measurements of the long bones of the limbs [7]. Ancestry was estimated by metric and non-metric assessments of the skull according to Navega et al. [8] and Hefner [9], respectively.

When estimating the postmortem interval (PMI), consideration was given to the presence or absence of soft tissue, structural breakdown of skeletal remains, and the local climate environment. To refine the range of time, we compared the records of previously identified remains examined by the LAF team in the past 25 years that were found in similar environmental conditions, considering that the time of the person’s disappearance is closely related to the date of death.

### Case 1

**Figure 1.**
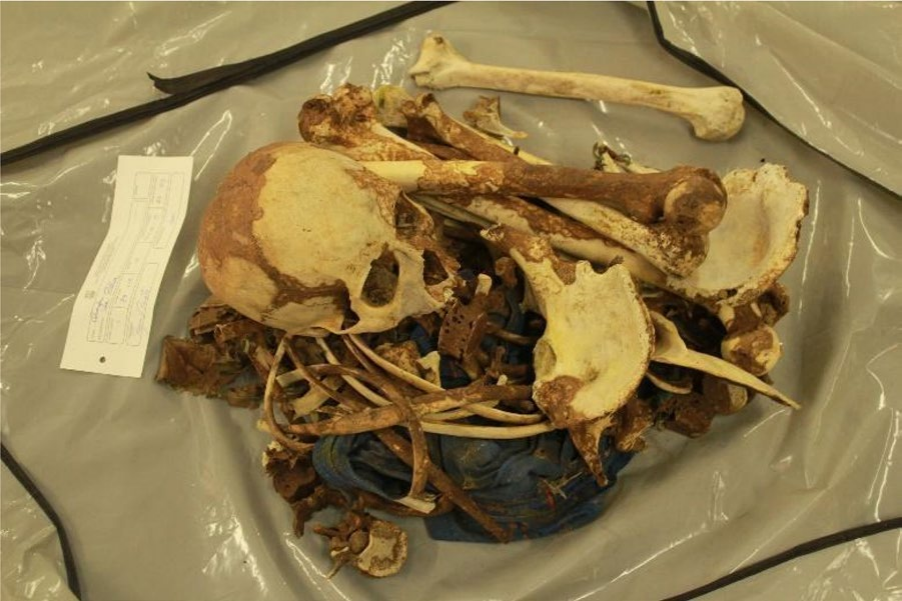
Skeletal remains as they were found in Case 1.

In June 2015, a human skeleton was admitted to the LAF for examination. Statements in the case history reported that a passer-by contacted police after finding human bones scattered over an area with an approximately 20-m radius in a rural region of the Federal District, Brazil. In accordance with internal protocol, a team from the LAF went to the site the following day. After a thorough scan of the area, they located and collected teeth and numerous bone pieces, including the jaw. The inventory showed an incomplete human skeleton totally devoid of soft parts and with bones showing whitish or brown surfaces caused by clay dirt. The bones generally showed areas of destruction consistent with chemical and/or physical actions of the environment where they were found, notably aggression by the cadaveric macrofauna (Figure 1). Garments in poor condition, including a shirt and shorts, were also mixed with the human remains. There was no evidence suggesting a possible identity of the deceased.

**Figure 2.**
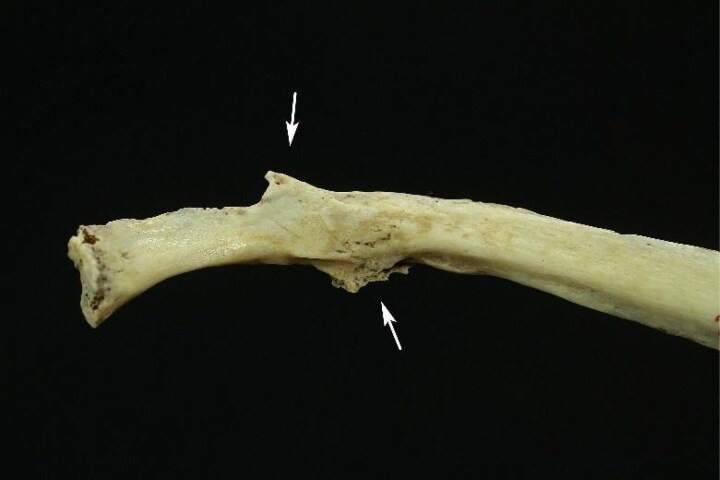
Sequelae of fracture in the 10th left costal arch (white arrows).

**Figure 3.**
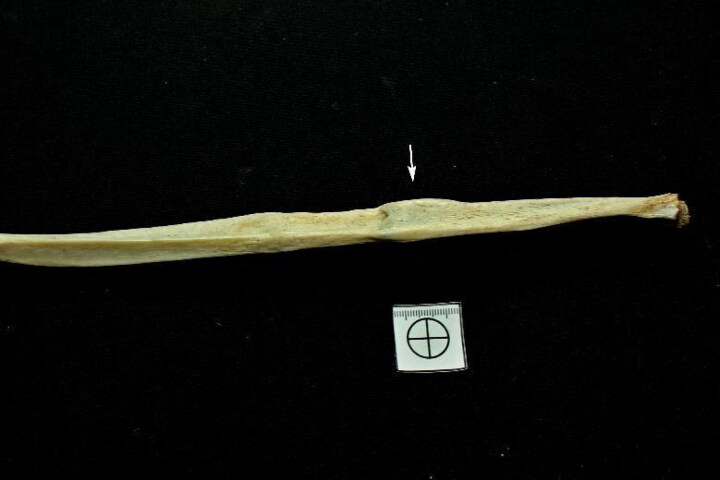
Sequelae of fracture in the left fibula (white arrow)
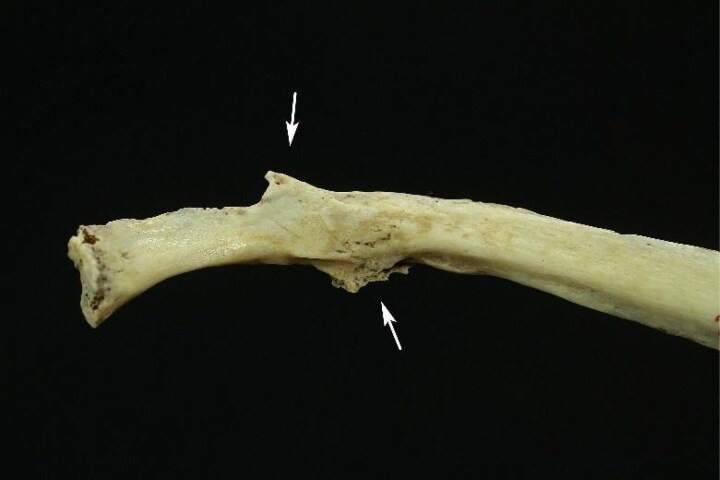
.

The bioanthropological profile showed a male, aged between 35 and 45 years, height between 1.61 and 1.69 m, with a probable triple ancestral contribution (European, African, and Amerindian). In Case 1, partial destruction of the pelvis, including the pubic symphysis of the fourth rib joint surfaces bilaterally and six out of 12 long limb bones, limited the use of all methods described above. However, this did not substantially impair the sex, age, and height estimates. The PMI was estimated to be greater than 1 year. The experts found no perimortem bone evidence that could clarify the cause of death. However, the left tenth rib and left fibula exhibited a bone callus consistent with old blunt trauma (Figures 2 and 3).

**Figure 4.**
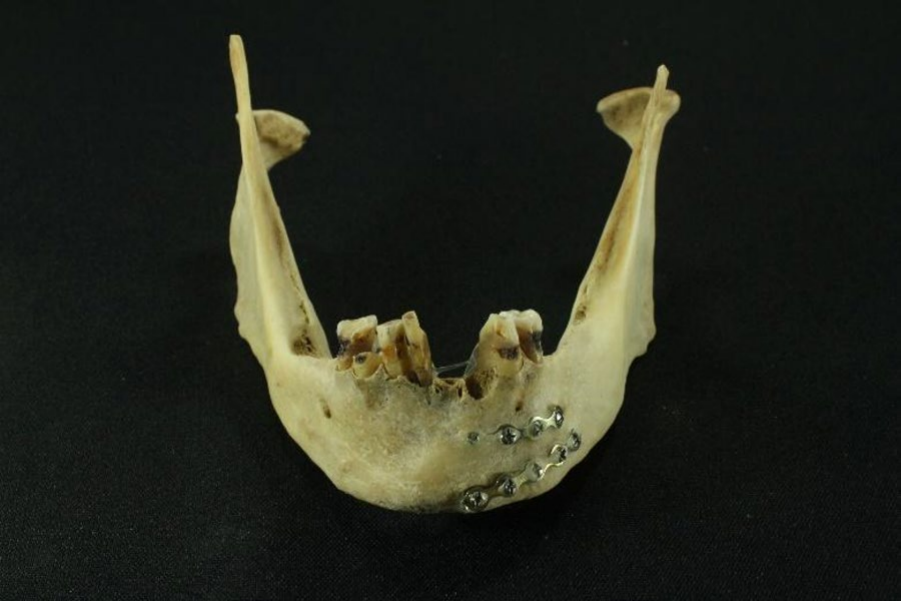
Jaw showing metallic osteosynthesis composed of two Erich bars and poorly maintained teeth.

The dental arches showed teeth in poor condition. The jaw showed signs of an old surgical procedure, more precisely a metallic osteosynthesis composed of two Erich bars used to fix a fracture on the left side of the body of the bone between the canine and the first premolar (Figure 4). This type of implant does not have a serial number, which makes it impossible to track by this parameter. The mandibular fracture was completely consolidated. Once the analysis was completed, no LAF investigators nor the police had any indication of the possible identity of the deceased despite observing various rare characteristics of the skeleton. A search of the missing person reports at the police station where the case was filed did not find a missing individual with a profile compatible with that of the deceased.

The poor state of conservation of the dental arches likely suggests an individual of low socioeconomic status in that jaw osteosynthesis is an uncommon surgical procedure. Given the evidence, we planned to search the files of the only local public hospital with an Oral and Maxillofacial Surgery Unit for any former patients who received such a surgical procedure and had a bioanthropological profile similar to that of the deceased. However, the search was not executed because the archive was paper-based, without clear organization, and contained thousands of files. Furthermore, we did not know when the jaw surgery was performed or if it was even performed at that hospital. The next step was to follow the usual procedure precluding the burial of an unidentified or unclaimed body. This procedure involves sharing the relevant data for identification in print and television media, which in this case were the location where the skeleton was found, the characteristics of the bioanthropological profile, the old fractures, the surgical procedure in the mandible, and the PMI. Despite this, no family member came forward to the police station or the FA laboratory to report a missing relative that fit these case details. The skeleton remains in the FA archive as unidentified.

### Case 2

**Figure 5.**
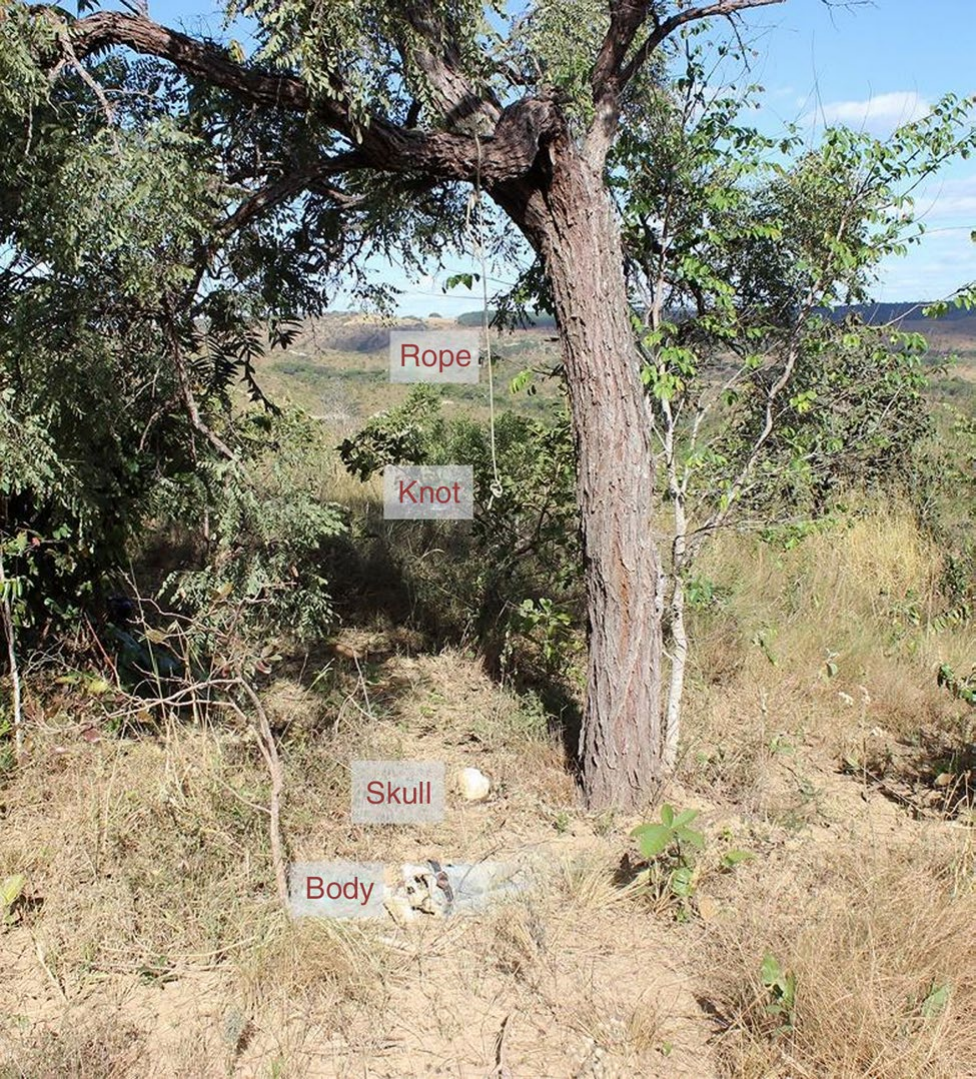
Hanging-compatible local scenario in Case 2.

**Figure 6.**
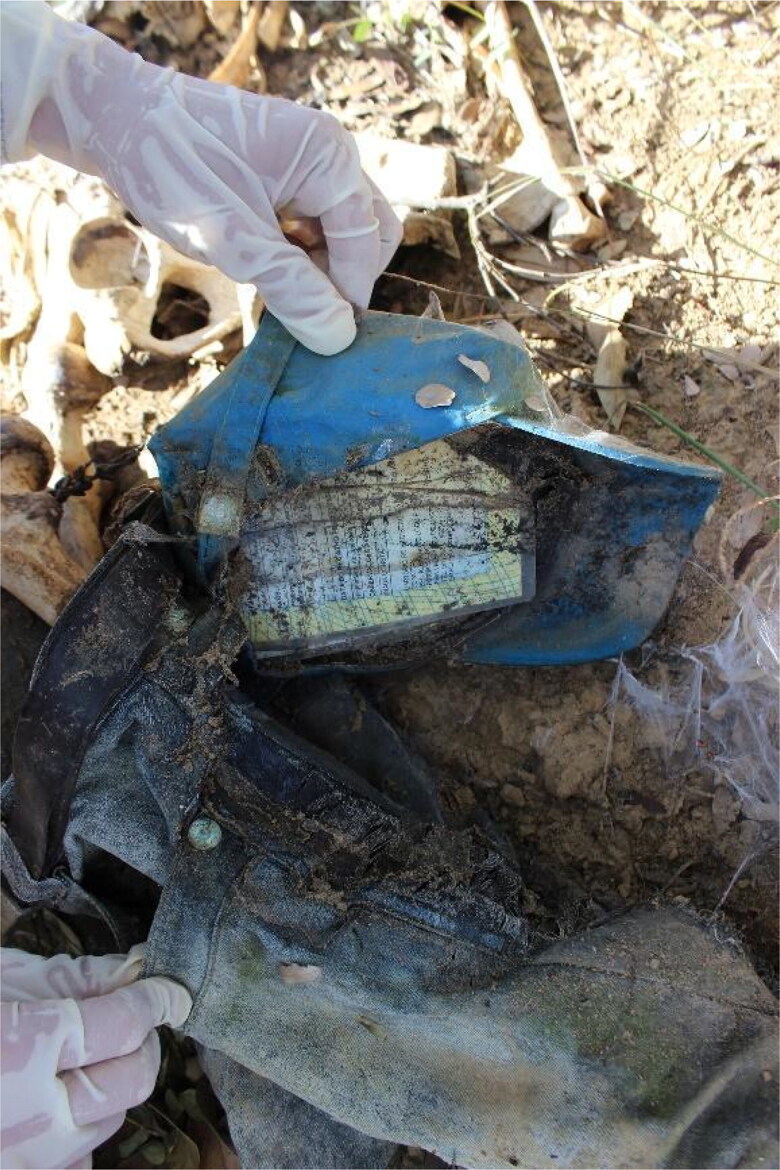
Cap tied to pants containing documents and other belongings.

In July 2016, human remains were found by a passer-by close to a highway in a rural area of the Federal District. The remains were located under a tree, from which a rope with a noose and a knot hung, constituting a gallows system (Figure 5). The human remains were dressed in frayed blue jeans, and the skull and jaw were disarticulated and resting approximately 1.5 m away from the body. A baseball cap containing a birth certificate, an identity card with a photo issued by the Identification Institute of the Federal District, and a National Health System card were tied to the pants (Figure 6). All documents contained the name of José Manoel dos Santos (fictitious name created by the author for this article) and included his date and place of birth and mother’s name. Inside the wallet, the experts also found a set of six 3 × 4 photographs depicting the same person on the identity card, a partially destroyed Brazilian currency banknote, two coins, and a piece of paper with “Dentist, Wednesday 05/07/14 PSF06, Bezerra” handwritten on it. The LAF travelled to the site where the remains were found and recovered some hand and foot bones, a tooth, and the hyoid bone missing the right major horn with no evidence of trauma.

Skeletal analysis showed compact bones with no cracks, a few of which were still connected by ligamentous and cartilaginous tissue, and scalp debris on the right side of the skull. The bioanthropological profile was consistent with a male, aged between 24 and 30 years, height between 1.66 and 1.74 m, and triple ancestral contribution (European, African, and Amerindian). The PMI was estimated to be between 3 and 12 months. No signs of skeletal trauma were found, and the scene was compatible with death by hanging.

The information in the suspect’s bioanthropological profile present in the Database of the Federal District’s Identification Institute, namely sex, age, and height, demonstrated consistency with the data obtained from the skeletal examination. By the time the investigation was completed, the police station had not yet located José’s family members, none of his family members attended the LAF, and his name was not in the police’s missing person list.

Considering that a bioanthropological profile and the documents and photographs found with the deceased were insufficient to provide a positive identification, other technical evidence was necessary. Police records showed that José had been an inmate at the local Specialized Youth Service Centre (CAJE), a juvenile prison, about 10 years earlier. We sent a letter to the CAJE explaining the case and requesting José’s family’s contact information, his dental records, and other information the CAJE officers believed may be useful for identification. However, CAJE replied that no matching records were found. The “PSF06” written on the paper in the pocket most likely stood for Health Office 06, a public health clinic in Formosa, a city in the neighbouring state of Goiás where José’s birth certificate was issued. Further attempts to find his dental records at the local public clinic were unsuccessful.

Despite these efforts, we were not able to contact José’s family nor find his dental records. Although the documents and bioanthropological profile pointed to José Francisco dos Santos, the identity remained only presumptive. The skeleton was then archived at the LAF awaiting additional information.

## Discussion

FA is responsible for performing forensic exams mainly on human bodies in an advanced state of decomposition or already completely skeletonized. The work normally involves addressing three fundamental points: (1) identification of the deceased, (2) cause of death, and (3) estimation of the PMI. Identification is always the most important.

Human remains can be identified by more than one means. The starting point for identification is usually the police investigation to determine the possible identity of the deceased and forward the relevant data to the forensic anthropologist. A suspected identity may be possible from the beginning when documents or other personal belongings are found with human remains. If these are not sufficient to identify the individual, they may constitute relevant information for identification. The success of identification by these means is contingent on finding family members or other people close to the individual who may provide antemortem (AM) material for comparison with postmortem (PM) data. Furthermore, identification can also be obtained without an initial suspected individual if one can rely on population databases of personal data such as fingerprints or missing person DNA. However, despite the Federal District’s extensive database of fingerprints, this was not useful in the present cases because of the complete decomposition of the respective digital pulps.

The failure to archive the identification in the two cases may also have been because both deceased people were potentially nomads, with no family members nor personal acquaintances noticing their disappearance. In the first case, the absence of a centralized missing person database within the PCDF increased the likelihood of failure in that the means by which we could match PM with AM data were limited. Because of this, the experts only searched the local database of the police station where the police report was filed; it was not feasible to search those of other police stations. However, this search did not yield any AM data compatible with the PM data.

Case 2 presents a different problem. Documents found with the human remains allowed for the collection of information about sex and age of the individual, while the civil record of the PCDF allowed us to later determine his height. This information was enough to establish the bioanthropological profile of the owner of the documents, which was compatible with the PM data. However, identification requires the use of at least one of the four primary methods of identification: pupilloscopy, forensic dentistry, forensic genetics, and comparative imaging [10]. There can be an exception in special situations when medical history may suffice, such as when they involve the presence of surgical implants [11] or complex tattoos [12]. However, none of these listed methods were available in the present case, so the identification could only be established as presumptive.

There is currently no Federal District or national missing person DNA database. To assist the federal government with its plans of creating such national databases of missing persons and unidentified human remains, the Brazilian Association of Forensic Anthropology initiated conversations with the Department of Justice and Public Security early in 2019 to lend its expertise to the initiative. Recently, one of the association’s board members, coming from one of the states that have already implemented a missing person database (the state of Minas Gerais), was appointed to a working group of Brazil’s Department of Justice and Public Security. The FA community eagerly awaits the fruits of the enterprise.
